# Mixed Central Sarcoma of the Left Humerus with Secondary Deposits in the Lung and Kidneys

**Published:** 1882-01

**Authors:** William Henry Porter


### CASE DEPARTMENT.

### I.—MIXED CENTRAL SARCOMA OF THE LEFT HUMERUS, WITH SECONDARY DEPOSITS IN THE LUNG AND KIDNEYS.

REPORTED BY WILLIAM HENRY PORTER, M.D., V.S.

THE specimen was presented at the New York Pathological Society, of New York, with the following history:

The animal afflicted with this large tumor was a fine mastiff. Some six or eight months prior to death the dog was chasing a cat, and ran with considerable force against a stone wall. When the owner of the animal reached the spot, the dog was lying near the wall howling pitifully, and could not be induced to move. She was accordingly wheeled home in a barrow. The injury was located at the left shoulder. Though very lame, she commenced using the limb after a few days, and at the end of a week or ten days had apparently recovered entirely. A week or two later, however, she again became lame, and a small tumor was noticed for the first time at the point of the left shoulder. The growth steadily increased in size, lameness became more and more marked, until finally the limb was useless as a means of support, and she steadily lost flesh and strength. When first seen by Dr. Frank V. Walton, House Surgeon of the Columbia Veterinary College Hospital, to which she was admitted, the animal was walking on three legs and drawing the toe of the left fore-leg on the ground. There was a large solid mass covering in the shoulder-joint and extending backwards so that the outlines of the scapula and humerus could not be detected. This growth was surrounded by considerable œdematous swelling and œdema of the left leg.

A diagnosis of traumatic aneurism was at first entertained, but later it was thought to be a sarcoma of some kind. The animal continued to lose flesh and strength, although her appetite remained good.

On April 9th, 1881, an attempt was made, under ether, to remove the growth, but the animal died under the operation.

The limb was then removed entire, when it was found that the growth seemed to spring from the humerus at the junction of the upper and middle thirds, at which point the bone was broken. From this central point the neoplasm radiated out in a centripetal manner, as is common in the periostial sarcomata. The tumor, which was surrounded by a thin fibrous capsule, extended above the superior end of the humerus, reaching into the infra spinous fossa. The growth was about nine inches long and five in diameter; at its superior extremity there were two large cysts containing bloody fluid. These cysts had smooth walls like the inside of a blood vessel, but careful examination failed to detect any communication with the blood vessels. There was also a small spiculæ of bone in the mass of the tumor, but none in the capsule. From this fact, together with the changes in the humerous, the growth was regarded as a central and not a periostial growth.

Examination of the internal organs revealed secondary deposits in lungs, kidneys and in the spleen; the liver was free from any secondary deposits.

The humerus, both above and below the fracture, was condensed, and the medullary cavity was completely obliterated. That portion of the infraspinous fossa against which the tumor rested was thickened and roughened, covered by numerous bony projections. After macerating the scapular all its surfaces were found covered by rounded bony projections, varying in size from a pin head to that of a small marble. The secondary deposits in the internal organs give good ground for believing that those on the scapular are sarcomaous and not simply inflammatory in origin.

*Microscopical examination* was made at the School of Histology, in connection with the Columbia Veterinary College and School of Comparative Medicine, by Prof. Wm. H. Porter. The tumor was found to be made up principally of various elements conforming to the connective-tissue type, the oval and round corpuscles being the predominating variety, though a few spindle and myeloid bodies were also noted. The secondary deposits were entirely of the round-cell variety.

The case is of interest, because it is probably the first one of the kind in the dog on record, although this form of malignant growth has been met with in others of the lower animals. Second, in adding still further proof that the disease of man and animals are quite similar. Third, that the growth seemed to follow directly after the receipt of severe injury. Fourth, it is of interest in connection with a case of secondary carcinoma of the kidney in a human subject, presented by Dr. Wendt at the Pathological Society, in which the secondary deposits were superficial in the kidneys, the same being true in all the organs in this case. It is also of interest in connection with the case of sarcoma of kidney in the cow, previously reported in this journal.

